# Salvage laryngectomy after primary radio- and radiochemotherapy: A retrospective study

**DOI:** 10.1007/s00106-021-01030-3

**Published:** 2021-05-21

**Authors:** Matti Sievert, Miguel Goncalves, Benedicta Binder, Sarina K. Mueller, Robin Rupp, Michael Koch, Stephan Dürr, Maximilian Traxdorf, Markus Hecht, Heinrich Iro, Antoniu-Oreste Gostian

**Affiliations:** 1 Department of Otorhinolaryngology, Head and Neck Surgery, Friedrich Alexander University of Erlangen-Nuremberg, Waldstraße 1, 91054 Erlangen, Germany; 2 Department of Radiation Oncology, Friedrich Alexander University of Erlangen-Nuremberg, Erlangen, Germany

**Keywords:** Recurrence, Salvage therapy, Laryngeal neoplasms, Treatment outcome, Postoperative complications

## Abstract

**Background:**

Recurrent and residual laryngeal cancer after organ-preserving radio- or radiochemotherapy is associated with a poor prognosis. Salvage surgery is the most important therapeutic option in these cases.

**Objective:**

The study assessed rates of recurrence and residual tumor as well as survival and complication rates after salvage laryngectomy at the authors’ academic cancer center.

**Materials and methods:**

A retrospective examination of all patients receiving laryngectomy between 2001 and 2019 due to tumor residuals or recurrence after primary radio- and radiochemotherapy was conducted.

**Results:**

A total of 33 salvage procedures were performed. Defect reconstruction was performed by free flap surgery in 30.3% (*n* = 10) and regional flap surgery in 15.2% (*n* = 5) . One patient received regional flap surgery and free flap surgery simultaneously. Overall survival after 1, 2, and 5 years was 68.7, 47.9, and 24.2%, and disease-free survival was 81.6, 47.8, and 24.2%, respectively, with 48.5% (*n* = 16) postoperative tumor recurrences overall. Disease-free survival was significantly shorter for tumor extension into or onto the hypopharynx (*p* = 0.041). Postoperatively, 72.7% of patients developed a pharyngocutaneous fistula, of which 24.2% required surgical treatment. The hospital stay was 28.0 ± 16.1 days.

**Conclusion:**

Salvage laryngectomy is associated with a high rate of treatable complications and high morbidity. Nevertheless, considering the advanced tumor stages treated, it allows for respectable oncological results.

**Supplementary Information:**

The online version of this article (10.1007/s00106-021-01030-3) includes the patient cohort. Article and supplementary material are available at www.springermedizin.de. Please enter the title of the article in the search field, the supplementary material can be found under “Ergänzende Inhalte”.

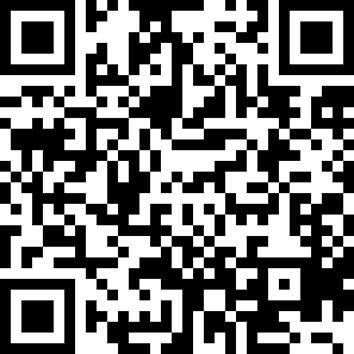

Salvage laryngectomy is a potential therapeutic option in treating recurrent and residual laryngeal cancer after radio- and radiochemotherapy. Under ongoing competition with and rapid advancement of nonoperative treatment methods for head and neck malignancy in recent years, an assessment of oncologic outcomes and complications of salvage surgery is essential for clinical decision-making.

## Background

Despite an increasing tendency towards primary organ-preserving strategies for locally advanced squamous cell carcinoma of the larynx and hypopharynx, salvage surgery remains of great importance. Depending on the primary tumor’s location and stage in the head and neck region, recurrence rates range from 25–50% after primary organ-preserving therapy [1]. In locoregional recurrence treatment, repetitive radiotherapy is often precluded, so effective oncologic outcomes can be achieved with salvage surgery [2]. After radiotherapy and chemotherapy, wound healing is impaired due to tissue fibrosis and decreased perfusion [3, 4]. This results in a significantly increased complication rate of up to 60% associated with markedly increased mortality and morbidity and significantly impaired quality of life [5]. The acceptance of the known disadvantages and risks of salvage surgery can only be justified by improved patient survival. Nowadays, immunotherapy is available as an alternative to surgery after failure of first-line therapy, in addition to palliative chemotherapy and re-irradiation [6]. Numerous clinical trials investigate the role of checkpoint inhibitors as a single treatment option and in combination with established treatment modalities. The current paradigm shift in the treatment of recurrent advanced laryngeal carcinoma puts the value of salvage surgery under critical review. This study aimed to determine the survival rate and evaluate preoperative prognostic factors for overall and disease-free survival of salvage surgery on residual or recurrent laryngeal cancer based on patients treated at the authorsʼ institution. Secondary objectives included evaluation of surgical and general postoperative complications and duration of tube feeding.

## Study design and methods of investigation

This is a retrospective cohort study of the University Hospital Erlangen (Department of Otolaryngology, Head and Neck Surgery), Germany. The study was approved by the local ethics committee (vote 246_20 Bc) and conducted in accordance with the Declaration of Helsinki.

### Study design

The analysis was performed using a retrospective review of records from the tumor database at the authorsʼ center of patients that underwent total or partial laryngectomy for laryngeal carcinoma between March 2001 and October 2019. Inclusion criteria were residual tumor or local recurrence after primary organ-preserving nonsurgical therapy. Residual tumor was defined as a residual tumor portion that was macroscopically and histologically confirmed on panendoscopy immediately after primary radio- or radiochemotherapy. Recurrence was defined as tumor recurrence after a disease-free interval and inconspicuous panendoscopy following radio- and radiochemotherapy. Patients that were in the disease stage of distant metastasis or had unresectable tumors were excluded. After extirpation of the larynx and partial laryngectomy with or without concomitant pharyngectomy, the defects were primarily closed or reconstructed with regional or free microvascular flap reconstruction, depending on their size. Percutaneous endoscopic gastrostomy (PEG) placement was performed as a prophylactic measure. A Gastrografin swallow was performed 10 days postoperatively. Oncological parameters (tumor, node, metastasis [TNM], resection [R] status), postoperative complications, and Eastern Cooperative Oncology Group (ECOG) status were documented. The disease stage classification was based on the 8th version of the Union Internationale Contre le Cancer (UICC, [7]).

### Outcome parameters

The study’s primary endpoints were oncologic outcomes with rates of local and regional recurrence and distant metastases, as well as disease-free survival and overall survival. Survival was defined from the date of surgery to the date of death from any cause (overall survival), the occurrence of recurrence (disease-free survival), or the date the patient was last known to be alive (overall survival and disease-free survival) or not dead from disease (disease-free survival). Patients that were alive at the time of evaluation were censored. Secondary endpoints represent the rate of postoperative complications, duration of tube feeding via PEG, and feeding at last follow-up.

### Statistical analysis

The metric parameters are presented with the mean and standard deviation (SD). Frequencies of variables are presented in absolute and relative values (*n*; %). Survival curves were generated using Kaplan-Meier estimation and compared using the log-rank test. Associations between individual anatomic locations with overall survival and disease-free survival were tested on univariate Cox models. The association between nominal variables was tested using the chi-square test. A *p*-value less than 0.05 was considered statistically significant. For statistical analysis, IBM SPSS Statistics, version 25.0 (IBM Corporation, Armonk, NY, USA), was used.

## Results

### Patient characteristics

A total of 1327 squamous cell carcinomas of the larynx were diagnosed at the authorsʼ center during the period in question: 1134 patients received primary surgical therapy, and 193 patients received primary organ-preserving therapy. Of these, 33 patients fulfilled the inclusion criteria (six females, 27 males; mean age, 63.9 ± 10 years, Table 1). First-line therapy was intensity-modulated radiotherapy (up to 67.3 ± 3.9 Gy total dose) in eight cases (24.2%). Simultaneous radiochemotherapy (up to 69.4 ± 3.4 Gy total dose; cisplatin, 5‑fluorouracil, docetaxel) was administered in 25 cases (75.8%). Eight patients received prior induction chemotherapy.

**Table 1:** Patient characteristics before primary therapy and before salvage surgery

*Gender*	*n (%)*	
Male	27 (81.8)	–
Female	6 (18.2)	–
**Variable**	**Initial**	**Salvage**
Age	Mean ± SD	Mean ± SD
Years	61.2 ± 10	63.9 ± 10
*T‑Stage*	*n (%)*	*n (%)*
T1	1 (3.0)	5 (15.1)
T2	9 (27.3)	4 (12.1)
T3	12 (36.4)	9 (27.3)
T4a	11 (33.3)	12 (36.4)
T4b	–	3 (9.1)
*N‑Stage*	*n (%)*	*n* *=* *17 (%)*^a^
N0	16 (48.5)	15 (88.2)
N1	4 (12.1)	–
N2b	7 (21.2)	–
N2c	6 (18.2)	–
N3b	–	2 (11.8)
*UICC*	*n (%)*	*n (%)*
I	1 (3.0)	5 (15.2)
II	4 (12.2)	4 (12.1)
III	11 (33.3)	7 (21.1)
IVa	17 (51.5)	12 (36.4)
IVb	–	5 (15.2)
*Grading*	*n (%)*	*n* *=* *31 (%)*
G1	–	1 (3.2)
G2	15 (45.5)	10 (32.3)
G3	18 (54.5)	20 (64.5)
*ECOG*	*n* *=* *32 (%)*	*n (%)*
ECOG 0	9 (28.1)	4 (12.1)
ECOG 1	13 (40.7)	9 (27.3)
ECOG 2	8 (25.0)	15 (45.5)
ECOG 3	1 (3.1)	4 (12.1)
ECOG 4	1 (3.1)	1 (3.0)

*UICC* Union Internationale Contre le Cancer, *ECOG* Eastern Co-operative Oncology Group Performance Status, *T* tumor, *N* node, *G* grade, *ND* neck dissection

^a^No ND in 16 cases

In the case of tumor recurrence (*n* = 12; 36.4%), salvage surgery was performed after a mean of 7.2 ± 3.8 months. Patients with local recurrence (*n* = 20; 60.6%) underwent surgery at a mean of 25.4 ± 18.1 months after initial diagnosis. One patient (3%) developed recurrence after a tumor-free interval of 27 years. Table 1 shows patient and treatment characteristics at the time of initial diagnosis and salvage surgery. An overview of the patient cohort can be found in the Electronic Supplementary Material online.

### Salvage surgery

A total of 33 salvage procedures were performed by complete (*n* = 31) or partial (*n* = 2) laryngectomy. Tumor-free margin (R0) could be achieved in a total of 30 cases (90.9%). An R1 situation was found in three patients (9.1%). In two cases, the R0 status initially diagnosed by intraoperative frozen section was R1 by the final histopathologic evaluation. In one patient, infiltration of the prevertebral fascia was detected intraoperatively. Neck dissection (level II–V) was performed in 17 cases (51.5%) simultaneously with salvage surgery (14 bilateral, three unilateral, Table 2). Positive neck status (ycN+) was diagnosed preoperatively on computed tomography in six patients (18.2%) and was confirmed histopathologically in two cases (6.1%). Patients were reclassified according to the histologic extent of tumor recurrence/residue (rpT). Pathologic reclassification revealed: five (15.1%) rpT1, four (12.1%) rpT2, nine (27.3%) rpT3, 15 (45.5%) rpT4 (Table 1). Pharyngeal reconstruction was performed by primary closure in 19 cases (57.6%). Due to larger resection defects, free microvascular flap reconstruction was performed in 10 patients (30.3%) and regional flap reconstruction was performed in five patients (15.2%) using pectoralis major myocutaneous flap (*n* = 2) or fasciocutaneous deltopectoral flap (*n* = 3) (Table 2). Of these, one patient received free as well as pedicled flap reconstruction. A total of 28 patients (84.8%) received PEG as part of therapy (Table 3). To achieve voice rehabilitation, six patients (18.2%) underwent primary tracheoesophageal puncture, and one patient (3.0%) underwent secondary tracheoesophageal puncture with the placement of a voice prosthesis (Provox 1, 2, and Vega; Atos Medical, Malmö, Sweden).

**Table 2:** Characteristics of treatment

*Primary therapy*	*n*	*%*
Radiotherapy	8	24.2
Radiochemotherapy^a^	8	75.8
Induction^b^	8	24.2
*Irradiation dose (Gray)*	*Mean*	*SD*
Tumor region	68.9	3.6
Lymphatic drainage basin	58.2	6.2
*Time span (irradiation–surgery)*	*Mean*	*SD*
Months	18.6	16.9
*Indication for salvage laryngectomy*	*n*	*%*
Residuum	12	36.4
Recurrence	20	60.6
Second malignancy	1	3.0
*Resection status*	*n*	*%*
R0	30	90.9
R1	3	9.1
*Pharyngeal reconstruction*	*n*	*%*
Primary suture	19	57.6
Pedunculated flap^c,d^	5	15.2
Free flap^e^	10	30.3
*Neck Dissection*	*n*	*%*
Unilateral	3	9.1
Bilateral	14	42.4

*R* resection, *SD* standard deviation

^a^Simultaneous radiochemotherapy

^b^Induction chemotherapy (cisplatin, 5‑fluorouracil, docetaxel)

^c^Patient #15 underwent pharyngeal reconstruction with one free and one pedicled flap

^d^Three deltopectoral flaps and two pectoralis major flaps

^e^Four anterolateral femoral flaps and six radial flaps

**Table 3:** Postoperative complications

*Surgical complications*	*n (%)*	*Duration in days (Mean* *±* *SD)*
Fistula without surgical revision	16 (48.5)	46.9 ± 35.6
Fistula with surgical revision	8 (24.2)	201.0 ± 170.9
Esophagotracheal fistula	1 (3.0)	–
Wound healing disorders	14 (42.4)	–
*Medical complications*	*n (%)*	*–*
Pulmonary artery embolism	1 (3.0)	
Stroke	2 (6.1)	
*Duration of inpatient stay*	*–*	*Duration in days (Mean* *±* *SD)*
		28.0 ± 16.1
*Duration of stay in intensive care unit*	*–*	*Duration in days (Mean* *±* *SD)*
		6.0 ± 5.8
*PEG Supply*	*n (%)*	*Duration in weeks (Mean* *±* *SD)*
Total	28 (84.8)	39.7 ± 20.8
Before salvage surgery	19 (57.6)	
At salvage surgery	8 (24.2)	
After salvage surgery	1 (3.0)	
*Nutrition at last presentation*	*n* *=* *32 (%)*	*–*
Completely via PEG tube	11 (34.4)	
Partially via PEG tube	5 (15.6)	
Normal or soft food (= PEG removed)	16 (50.0)	

*PEG* percutaneous endoscopic gastrostomy

### Oncological outcome

**Fig. 1:**
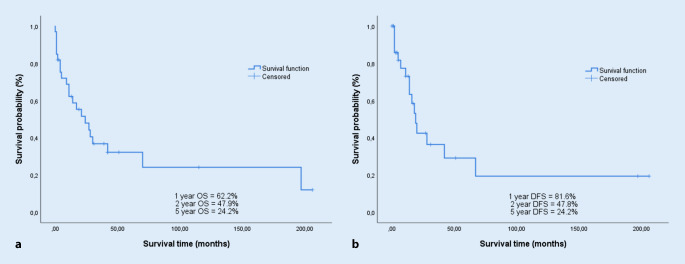
Overall survival (**a**) and disease-free survival (**b**)

Of 33 patients, 16 (48.5%) presented with tumor recurrence after salvage surgery. Local recurrence was observed in nine patients (27.3%) after a mean of 83.4 ± 87.8 weeks, and regional cervical recurrence was observed in three patients (9.1%) after 159.6 ± 121.2 weeks. Distant metastases were diagnosed in 11 patients (33.3%) after a mean of 76.36 ± 80.6 weeks. The 1‑, 2‑, and 5‑year overall survival rates were 68.7%, 47.9%, and 24.2%, respectively, with a median survival of 11 months (0–206 months). Disease-free survival at 1‑, 2‑, and 5‑years is 81.6%, 47.8%, and 24.2%, respectively (Fig. 1). In univariate regression analysis, tumor manifestation in the hypopharynx (*p* = 0.033) was revealed to be a significant negative predictor of overall survival. Disease-free survival was significantly reduced in the case of tumor manifestation in the hypopharynx (*p* = 0.041).

### Postoperative outcome

An overview of all surgical and medical postoperative complications is presented in Table 3. Complications occurred in a total of 25 patients (75.7%). Pharyngocutaneous fistula was the most common complication at 72.7% (*n* = 24). In 16 cases (48.5%), the fistula occluded following purely conservative treatment after an average of 46.9 ± 35.6 days. Eight patients (24.2%) required surgical fistula closure by pectoralis major or deltoid flap. No association was found between patients with and without primary flap reconstruction in terms of fistula rate (*p* = 0.618). The patient with the secondary placement of a Provox prosthesis developed an extensive tracheoesophageal fistula. Patients were hospitalized for a mean of 28.0 ± 16.1 days, including 6.0 ± 5.8 days in the intensive care unit. After an average of 34.0 ± 50.8 months at last follow-up, 16 patients (50.0%) were feeding entirely orally. The PEG was removed in these patients after an average of 39.7 ± 20.8 weeks. Five patients (15.6%) were partially dependent on the PEG at the last presentation, and 11 patients (34.4%) were completely PEG-dependent (Table 3).

## Discussion

The present study assesses the outcome of patients undergoing salvage surgery by total or partial laryngectomy after primary nonsurgical treatment. The results show a 2- and 5‑year overall survival of 47.9% and 24.2%, respectively (Fig. 1), and a disease-free survival of 47.8% and 24.2%, respectively. Overall, 48.5% of patients had tumor recurrence postoperatively. A significantly lower 5‑year survival rate was observed in the presence of hypopharyngeal involvement (28.8% versus 10.9%; *p* = 0.041). Salvage surgery was associated with a high complication rate. Pharyngocutaneous fistula was the most common complication, requiring mostly conventional therapy only. In all, 50% of the treated patients were able to receive complete oral nutrition postoperatively.

In general, the rate of locoregional recurrence after organ-preserving therapy is 30–50% [1, 8]. If there is residual tumor or locoregional recurrence after primary radiochemotherapy, salvage surgery options should be explored. It remains a curative form of treatment after failure of primary nonsurgical therapy, provided that complete resection with negative margins appears achievable preoperatively. More than 70% of patients with residual disease or recurrence after radiochemotherapy have locally advanced tumors in the T3 and T4 categories [9]. The authors can confirm this with 27.3% of patients in an early (UICC I and II) and 72.7% of patients in a locally advanced tumor stage (UICC III and IV). In consideration of the study period and the development in radiation technology therein, the patients treated with salvage surgery in the context of the present study are comparable to the data of Putten et al. [5] for the study years 1990–2007. Thus, salvage surgery remains an ongoing surgical treatment option for the oncologic surgeon, albeit challenging and associated with complications. Overall oncologic outcomes across therapeutic regimens are unsatisfactory. In summary, the overall survival of 48–49% is reported for purely laryngeal carcinomas and 17–26% with hypopharyngeal involvement [1, 10]. Alternatively, repeat radio- or radiochemotherapy may be offered in curative intent. A comprehensive meta-analysis on the outcomes of re-radiation was recently published by Grün et al. These colleagues reported 2‑ and 5‑year overall survival rates of 47–57% and 23%, respectively, after intensity-modulated radiotherapy with concomitant chemotherapy [11]. Of note are the associated side effects, some of which are severe, with acute severe reactions in up to 73%. Acute life-threatening complications are described in up to 11% [11, 12]. According to Kowalski et al., palliative therapy alone resulted in a median survival of only 3.8 months in 797 patients with recurrent head and neck malignancy and should be recommended with caution if operability is possible [13].

Difficult intraoperative preparation due to scarring post-radiogenic changes and reduced tissue perfusion is characteristic of salvage surgery [2]. This causes significantly increased postoperative complication rates with wound healing disorders, lymphedema, and especially the formation of pharyngocutaneous fistulas, which is the most common surgical complication [4].

Conservative therapy is the primary treatment in these cases, so surgical treatment is necessary for only about one-third of all fistulas [4, 14, 15]. In particular patients with a fistula requiring revision showed a prolonged healing course with a significant reduction in quality of life [15]. For example, the reconstruction of the pharynx with fresh tissue, for example, by pectoralis major flap, has a protective effect on the fistula rate [15]. In terms of the expected complications after salvage surgery and maintaining an acceptable quality of life and nutrition, PEG placement also contributes here [16, 17]. Two-thirds of patients were only partially, or not at all, dependent on tube feeding at their last presentation, after an average of 25 months.

Due to the problem of preoperative “understaging” caused by post-radiogenic edema and scaring changes, as well as the often multicentric tumor sites of recurrences, total laryngectomy remains the preferred procedure in salvage surgery of laryngeal carcinoma [10, 18, 19]. The results of this rare and challenging therapy must be interpreted in light of the retrospective study design and the inevitable limitations associated with it. This also results in a limited number of patients, which corresponds to comparable studies on this topic but impairs the analysis of individual factors influencing the oncological outcomes considered. Another aspect is the considerable period of retrospective data collection, which includes significant developments in radiation oncology. Nevertheless, the characteristics of patients to be treated are comparable to those in the 1990s and early 2000s. Salvage surgery for laryngeal cancer remains a treatment option that still retains its value today over alternative re-irradiation options and should be discussed in detail with the patient in these challenging situations.

## Practical conclusion

- Salvage laryngectomy remains the current best therapeutic strategy in curative intent for residuals and recurrences after organ preservation protocols and should be considered as a therapeutic option.
- In particular, patients with small, strictly laryngeal tumors without cervical metastasis benefit from salvage laryngectomy.
- Complications, especially pharyngocutaneous fistulas, are frequent but can be successfully treated conservatively.

## Supplementary Information

Overview of the patient cohort.

## Declarations

This retrospective study was performed after consulting the relevant ethics committee and in accordance with national law, as well as the Declaration of Helsinki.

The supplement containing this article is not sponsored by industry.
