# Supplementary material for: Salvage laryngectomy after primary radio- and radiochemotherapy: A retrospective study
Source: HNO. 2021 May 21;69(Suppl 2):47–52. doi: 10.1007/s00106-021-01030-3 (PMC8397647; doi:10.1007/s00106-021-01030-3)
Supplement: Supplementary file 1 — Overview of the patient cohort. [file 106_2021_1030_MOESM1_ESM.pdf]

Overview of the patient cohort

Nr.	G	A (ID)	TR	cT	cN	UICC (ID)	PT	TG (Gy)	yrcT	yrcN	OP	A (OP)	ND	Reko	rpT	rpN	UICC (OP)	R	Rez	tR (W)	Status	ST (M)	DFS (M)
1	m	42	La	cT2	cN1	III	RCT	65,0	T2	N0	kLE	70	-	-	pT1		I	R0	-		A		2,0
2	w	67	HP	cT4a	cN2b	IV	RCT	72,0	T4a	N1	kLE	70	b	Ra	pT3	pN0	III	R0	-		A		3,0
3	m	59	La	cT3	cN2c	IV	RCT	66,0	T4a	N2b	kLE	59	b	ALT	pT3	pN3b	IV	R0	-		A		13,0
4	m	72	La	cT3	cN0	III	RCT	66,0	T3	N0	kLE	75	b	-	pT3	pN0	III	R0	-		D	4,0	4,0
5	w	51	La	cT4a	cN2b	IV	RT	68,0	T4a	N0	kLE	54	b	Ra	pT4a	pN0	IV	R0	l	82	A		18,0
6	m	55	La	cT3	cN0	III	RCT	72,0	T3	N0	kLE	56	-	-	pT4a		IV	R0	-		A		31,0
7	m	72	La	cT3	cN2b	IV	RCT	70,0	T3	N1	kLE	74	b	-	pT4b	pN0	IV	R0	f	12	D	4,0	2,0
8	m	55	La	cT2	cN2b	IV	RCT	72,0	T3	N0	kLE	56	b	-	pT3	pN3b	IV	R0	f	125	A		28,0
9	m	53	La	cT4a	cN0	IV	RCT	68,0	T4a	N0	kLE	55	-	-	pT3		III	R0	f	46	A		10,0
10	m	52	La	cT2	cN0	II	RCT	66,0	T3	N0	kLE	55	-	ALT	pT3		III	R0	l/r/m	62	A		14,0
11	m	62	HP	cT4a	cN2c	IV	RCT	70,0	T4a	N0	kLE	67	-	Ra	pT1		I	R0	f	88	D	28,0	20,0
12	m	49	La	cT2	cN2b	IV	RCT	72,0	T2	N0	kLE	49	b	-	pT2	pN0	II	R0	-		A		51,0
13	w	60	La	cT2	cN2b	IV	RCT	66,0	T2	N0	kLE	62	u	Ra	pT2	pN0	II	R0	f	33	D	42,0	7,0
14	m	82	La	cT4a	cN1	IV	RCT	72,0	T4a	N0	kLE	83	b	-	pT3	pN0	III	R0	f	11	D	2,0	2,0
15	m	69	HP	cT3	cN0	III	RCT	70,6	T4a	N0	kLE	69	b	Ra/DP	pT3	pN0	III	R0	-		D	1,0	1,0
16	m	60	HP	cT4a	cN2b	IV	RCT	70,0	T4a	N1	kLE	61	b	Ra	pT3	pN0	III	R0	r/m	61	D	14,0	14,0
17	w	65	HP	cT4a	cN0	IV	RCT	71,2	T3	N0	kLE	67	-	-	pT4a		IV	R1	-		D	1,0	0,0
18	m	58	HP	cT3	cN0	III	RCT	73,6	T3	N0	kLE	62	u	PM	pT4b	pN0	IV	R0	-		D	0,0	0,0
19	m	68	HP	cT2	cN0	II	RCT	72,0	T4a	N0	kLE	72	-	ALT	pT4a		IV	R0	-		D	1,0	1,0
20	w	54	HP	cT3	cN1	III	RCT	70,0	T4a	N0	kLE	55	u	ALT	pT4b	pN0	IV	R0	l	12	A		2,0
21	m	69	HP	cT2	cN2c	IV	RCT	71,8	T3	N0	kLE	71	-	PM	pT1		I	R0	-		D	27,0	27,0
22	m	49	HP	cT3	cN0	III	RCT	72,4	T3	N0	kLE	50	-	-	pT4a		IV	R0	l	85	D	24,0	19,0
23	m	62	La	cT4a	cN2c	IV	RCT	72,0	T4a	N1	kLE	63	b	-	pT4a	pN0	IV	R1	l	72	D	30,0	0,0
24	m	80	La	cT2	cN0	II	RT	70,2	T4a	N0	kLE	81	-	-	pT4a		IV	R0	l	49	D	11,0	11,0
25	m	86	La	cT1	cN0	I	RT	66,6	T2	N0	kLE	91	-	-	pT2		II	R0	-		D	11,0	11,0
26	m	78	La	cT3	cN0	III	RT	70,0	T4a	N0	kLE	81	-	DP	pT4a		IV	R1	-		D	5,0	0,0
27	m	68	La	cT3	cN1	III	RT	70,6	T4a	N0	kLE	68	-	DP	pT4a		IV	R0	l/m	22	D	9,0	5,0
28	m	51	HP	cT4a	cN0	IV	RCT	63,0	T4a	N0	kLE	54	b	-	pT4a	pN0	IV	R0	-		D	1,0	1,0
29	m	72	La	cT3	cN0	III	RT	70,2	T3	N0	kLE	72	-	-	pT4a		IV	R0	-		D	17,0	17,0
30	m	65	La	cT3	cN0	III	RT	64,0	T3	N0	kLE	70	-	-	pT4a		IV	R0	l/r/m	295	D	70,0	67,0
31	m	67	La	cT2	cN0	II	RT	59,4	T3	N0	kLE	69	b	-	pT2	pN0	II	R0	-		A		206,0
32	m	46	La	cT4a	cN2c	IV	RCT	60,0	T4a	N2b	PL	47	b	-	pT1	pN0	I	R0	-		D	197,0	197,0
33	w	63	La	cT4a	cN2c	IV	RCT	72,0	T4a	N0	PL	64	-	-	pT1		I	R0	l/m	11	D	21,0	2,0

Abbreviations:

G: Gender
A: Age
ID: Initial Diagnosis
TR: Tumor region
La: Larynx
HP: Hypopharynx
cT: clinical Tumor Classification

cN: clinical Lymph Node Classification
Ra: Radial forearm flap
PT: Primary Therapy
TG: Tumorgebiet
RCT: Radio-Chemotherapy
RT: Radiotherapy
Gy: Gray
yrcT: Tumor Extension after Primary Therapy
yrcN: Lymph node inolvement after Primary Therapy
kLE: Complex Laryngectomy

PL: Partial Laryngectomy

ND: Neck Dissection
b: bilateral
u: unilateral
Reko: Type of reconstruction
ALT: Anterolateral Thigh Flap
DP: Deltopectoral flap
PM: Pectoralis major flap

rpT: histopathological tumor extension at salvage surgery
rpN: histopathological lymph node involvement at salvage surgery
R: Resection status
Rez: Recurrence
l: Local recurrence
r: Regional recurrence
m: Distant metastases
A: Alive

D: Dead

ST: Survival Time (months)
DFS: Disease Free Survival

Type of distant metastases:

Nr. 7: mediastinal lymph node/pulmonary
Nr. 8: pulmonary
Nr. 9: pulmonary/soft tissue/osseous
Nr. 10: pulmonary
Nr. 11: pulmonary
Nr. 13: pulmonary l
Nr. 14: pulmonary
Nr. 16: pulmonary/hepatic/osseous
Nr. 27: skin
Nr. 30: pulmonary
Nr. 33: pulmonary
